# A systematic literature review and meta‐analysis on digital health interventions for people living with dementia and Mild Cognitive Impairment

**DOI:** 10.1002/gps.5730

**Published:** 2022-05-19

**Authors:** Claudio Di Lorito, Alessandro Bosco, Harleen Rai, Michael Craven, Donal McNally, Chris Todd, Vicky Booth, Alison Cowley, Louise Howe, Rowan H. Harwood

**Affiliations:** ^1^ School of Medicine University of Nottingham Nottingham UK; ^2^ School of Health Sciences University of Manchester Manchester UK; ^3^ Computer and Information Sciences University of Strathclyde Glasgow UK; ^4^ Mindtech, Institute of Mental Health Nottingham UK; ^5^ Faculty of Engineering University of Nottingham Nottingham UK; ^6^ Nottingham University Hospitals NHS Trust Nottingham UK; ^7^ School of Health Sciences University of Nottingham Nottingham UK

**Keywords:** dementia, digital health, effectiveness, information technology, literature review, meta‐analysis, Mild Cognitive Impairment, rehabilitation

## Abstract

**Objectives:**

Digital health interventions enable services to support people living with dementia and Mild Cognitive Impairment (MCI) remotely. This literature review gathers evidence on the effectiveness of digital health interventions on physical, cognitive, behavioural and psychological outcomes, and Activities of Daily Living in people living with dementia and MCI.

**Methods/Design:**

Searches, using nine databases, were run in November 2021. Two authors carried out study selection/appraisal using the Critical Appraisal Skills Programme checklist. Study characteristics were extracted through the Cochrane handbook for systematic reviews of interventions data extraction form. Data on digital health interventions were extracted through the template for intervention description and replication (TIDieR) checklist and guide. Intervention effectiveness was determined through effect sizes. Meta‐analyses were performed to pool data on intervention effectiveness.

**Results:**

Twenty studies were included in the review, with a diverse range of interventions, modes of delivery, activities, duration, length, frequency, and intensity. Compared to controls, the interventions produced a moderate effect on cognitive abilities (SMD = 0.36; 95% CI = −0.03 to 0.76; *I* ^2^ = 61%), and a negative moderate effect on basic ADLs (SMD = −0.40; 95% CI = −0.86 to 0.05; *I* ^2^ = 69%). Stepping exergames generated the largest effect sizes on physical and cognitive abilities. Supervised training produced larger effect sizes than unsupervised interventions.

**Conclusion:**

Supervised intervention delivery is linked to greatest benefits. A mix of remote and face‐to‐face delivery could maximise benefits and optimise costs. Accessibility, acceptability and sustainability of digital interventions for end‐users must be pre‐requisites for the development of future successful services.

## Key points

- Digital health interventions have enabled services to keep delivering health care to people living with dementia during the COVID‐19 pandemic, and while evidence on their effectiveness is mounting, an update of the literature is needed.
- This systematic review of the literature on digital health interventions for people living with dementia included 20 studies on a diverse range of interventions, modes of delivery, activities, duration, length, frequency, and intensity.
- Digital health interventions produced positive effects on cognitive abilities and negative effects on activities of daily living, compared to non‐digital interventions.
- Stepping exergames generated the largest effect sizes on physical and cognitive abilities, while supervised training produced larger effect sizes than unsupervised interventions.

## 1 INTRODUCTION

Digital health interventions, defined as “Applications, programmes and software used in the health and social care system”^1^ have taken centre stage during the COVID‐19 pandemic. Many of the elements enabling face‐to‐face health care became impossible to deliver when measures mandated by governments to slow the spread of the virus required older people with pre‐existing conditions to shield.^2^ Digital health interventions have enabled services to keep delivering health care to people remotely.

Evidence is mounting on the benefits of digital health interventions for people living with dementia and Mild Cognitive Impairment (MCI).^3^, ^4^, ^5^, ^6^ Digital technologies represent a viable option to support this population to combat their risk of apathy, social exclusion, sedentary lifestyles, to get active and engage in health promotion behaviours, thus potentially reducing injury and hospitalisation, and delaying access to care homes.^7^ They may also benefit people with dementia who struggle to engage in community programmes/activities because of mobility, social anxiety, accessibility issues and/or geographical isolation.^7^

Several digital health interventions for people living with dementia/MCI have been developed ex novo or adapted from a non‐digital form, to provide equitable services for people who cannot access community services, and particularly over the last 2 years, to ensure continuation of support/delivery during times of social distancing due to the COVID‐19 pandemic.^7^ From a service delivery perspective, there is also the rationale of potentially reducing costs through, for example, not needing to travel to service users' homes. A diverse range of digital health services have been developed and tested, including interventions providing cognitive stimulation,^8^, ^9^ exergaming,^10^, ^11^, ^12^ resources for the person and carers to address health care issues,^13^, ^14^ and in‐home technologies and/or live support for users.^15^ These interventions are typically complex, as they include a number of interacting components,^16^ which can be classified under the terms ‘design, content, and delivery features’.^17^ ‘*Design*’ is the mode of delivery (i.e., “how”, e.g. a virtual reality‐enhanced, recumbent stationary bike); ‘*content*’ is the materials, procedures, activities, and/or processes (i.e., “what”, e.g., participants pedal in 360‐degree radius to locate coloured dragons of varying speed); ‘*delivery*’ is about intervention implementation (i.e., “who, where, when, how much”, e.g., group/individual, location, duration, length, frequency and intensity).^18^

Studies during the COVID‐19 pandemic raised questions around the effectiveness of digital health interventions on clients with MCI/dementia,^7^ as generational barriers including computer literacy, and cognitive impairment‐specific difficulties such as memory problems or apathy may thwart intervention effectiveness. To date, a number of literature reviews have gathered evidence on the feasibility and efficacy of digital health interventions in a population with dementia^3^, ^14^, ^19^, ^20^, ^21^ and around its barriers and facilitators.^22^ However, to our knowledge, there is no published work comparing different types of digital health interventions. Further, given the everchanging evolution in the field of digital health caused by the COVID‐19 pandemic, an update of the literature is needed. This systematic review of the literature aims to gather updated empirical evidence on digital health interventions for people living with dementia/MCI. The objectives are:

1. To describe the *types of interventions, design, content, and delivery features;*
2. To *meta‐analyse reported effects (positive and negative)* on physical, cognitive, behavioural and psychological outcomes and Activities of Daily Living (ADLs);
3. To report the *positive effects* on outcome parameters;
4. To identify the interventions linked to *largest improvements* on outcome parameters.

## 2 METHODS

This work conforms with the Preferred Reporting Items for Systematic Reviews and Meta‐Analyses (PRISMA) Statement^23^ (Appendix A).

### 2.1 Search

The search strategy (Appendix B) was based on the PICO (Population, Intervention, Comparison, Outcome) worksheet for systematic reviews.^24^ It was developed by the research team and finetuned by a librarian from the University of Nottingham. The searches were run by one author (CDL) in November 2021 in nine databases: The Allied and Complementary Medicine Database (AMED), the Cumulative Index to Nursing and Allied Health Literature (CINAHL), the Cochrane Central Register of Controlled Trials (CENTRAL), Embase, Medline, PsycInfo, SportDiscus, Web of Science and Google Scholar.

### 2.2 Study selection and appraisal

All initial records were imported into Endnote and duplicates removed. Two authors (CDL and AB) separately carried out title and abstract screening, eliminated ineligible studies and then screened the full texts of the remaining records against the inclusion/exclusion criteria. Any disagreement was resolved by reaching consensus in a meeting between CDL and AB. A contingency plan was in place to involve a third adjudicating author (MC) in case consensus between CDL and AB was not reached. All disagreements were resolved through discussion without the need to involve the adjudicator. Numbers/reasons for exclusion were recorded. The references of the included studies were screened to identify further eligible studies.

#### 2.2.1 Inclusion criteria

- Randomised Controlled Trials (RCTs) and non‐RCTs (baseline vs. follow up and/or intervention vs. control) on physical and/or cognitive outcomes and/or behavioural and/or psychological outcomes.
- Evaluating any digital health intervention, defined as “Applications, programmes and software used in the health and social care system”^1^ developed for adults with dementia (any type)/MCI.
- Any publication year.
- Published in English.

#### 2.2.2 Exclusion criteria

- Studies without a control group.
- Studies where data were not presented separately for participants with MCI/dementia and those without.
- Interventions targeting caregivers only.
- Studies not report on effectiveness or having a positive effect on the outcomes of interest (physical and/or cognitive outcomes and/or behavioural and/or psychological outcomes).

### 2.3 Study quality appraisal

Two raters (CDL and AB) assessed the quality of the studies through the Critical Appraisal Skills Programme (CASP) checklist.^25^ The raters discussed each study and agreed on a final quality score. The CASP was used for quality screening purposes only and not to exclude any study on the grounds of poor quality (selection of study was strictly based on inclusion/exclusion criteria only). Because of the lack of reporting in the individual studies and of the subjectivity in attributing score, items 9, 10 and 11 of the CASP were operationalised as follows:

Item 9: “Do the benefits of the experimental intervention outweigh the harms and costs?” was operationalised as “Would the benefits reported in the study potentially outweigh costs associated with successful implementation of the digital intervention (e.g., development, commercialisation, accessibility)?”

Item 10: “Can the results be applied to your local population/in your context?” was operationalised as “Are the results generalisable to the diversity of people living with dementia (e.g., different stages of the condition, different socio‐economic status)”?

Item 11: “Would the experimental intervention provide greater value to the people in your care than any of the existing interventions?” was operationalised as: “Would the experimental intervention provide greater benefits than non‐digital version of the same intervention”?

The total possible score for the quality appraisal was 12, with higher scores showing higher quality. The raters agreed that when the study did not report information for an item, it would be rated negatively (i.e., ‘no’).

### 2.4 Data extraction and analysis

Study characteristics were extracted by the first author (CDL) using a modified version of the data extraction form in the Cochrane handbook for systematic reviews of interventions.^26^ Data on the design, content and delivery features of the interventions (see “Introduction” for definitions) were extracted using a modified version of the template for intervention description and replication (TIDieR) checklist and guide.^18^ The forms were first piloted on a random sample of three studies, and then used by the first author (CDL) to complete data extraction. When complete, the process was checked for accuracy by the second author (AB). The design, content and delivery features of interventions (Objective 1) and the effectiveness of interventions on physical, cognitive, behavioural and psychological outcomes and ADLs (Objective 2) were reported through deductive thematic analysis,^27^ with themes were established a priori.

For the meta‐analyses of effect sizes, we only included study with between‐groups (i.e., interventions vs. control) comparisons. We first considered heterogeneity of studies to decide if combining the results would be clinically meaningful using the *I* ^2^ statistic and the parameters provided in the Cochrane handbook for systematic reviews of interventions:^26^ 0%–40%: heterogeneity not important; 30%–60%: moderate heterogeneity; 50%–90%: substantial heterogeneity; 75%–100%: considerable heterogeneity. If the studies were considerably heterogeneous, we did not proceed with data pooling. Otherwise, we conducted meta‐analyses using a random‐effects model, and then performed sensitivity analyses through the leave‐one‐study method to identify whether any one study affected the pooled estimates. Standard Mean Difference (SMD) was used as metric of effect size in the meta‐analysis, using the parameters: 0.2–0.5: small; 0.5–0.8: medium, > 0.8: large. Meta‐analyses were performed using Review Manager (RevMan) V 5.4.1.

Identification of the interventions linked to largest improvements for each of the outcomes (Objective 4) was carried out by identifying effect sizes. Therefore, only studies reporting effect sizes were considered. Cohen's *d* was used as the unit measure of effect size. Effect sizes of studies using other measures (e.g., Odds Ratio) were converted into Cohen's *d* through the scales of magnitude by Cohen^28^ and Lu and Chen.^29^

## 3 RESULTS

**FIGURE 1:**
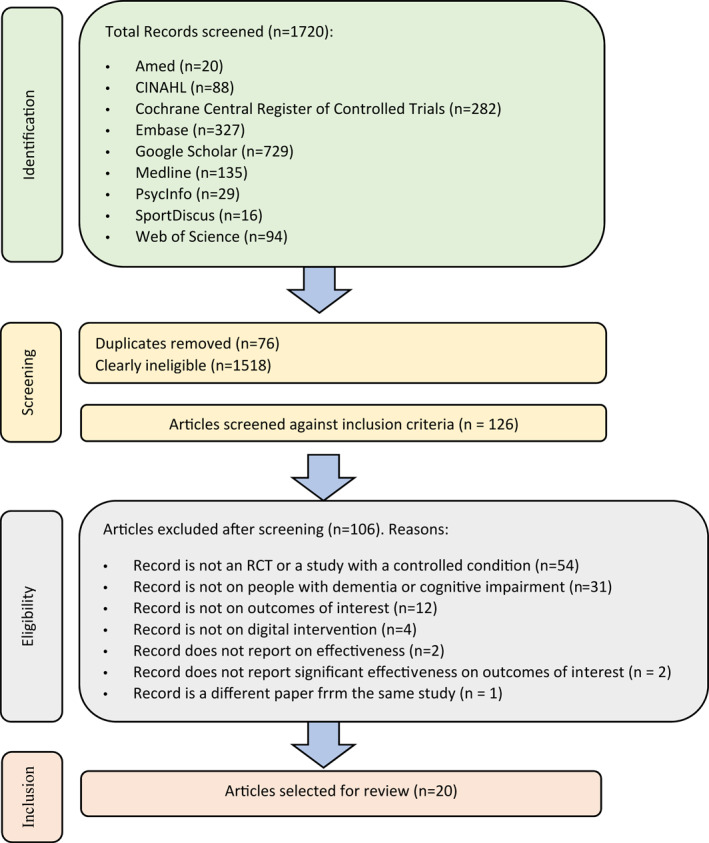
Selection of papers

The searches identified 1720 records (Figure 1). Of these, 202 passed title and abstract screening. Seventy‐six duplicates were removed and the full text of 126 remaining records was assessed against the inclusion/exclusion criteria. Of these, 106 were excluded. Twenty records passed the full text screening and were included in this review.

### 3.1 Study quality appraisal

All studies were rated positively on items 1 to 3 and were therefore included in the review based on their quality. The quality score ranged from eight to 12. The items most often rated ‘no’ were in relation to participant, investigator and assessor blinding to intervention. Precision of the estimate of the intervention (i.e., 95% confidence intervals) was also sparsely reported (Table 1).

**TABLE 1:** Study quality appraisal

	CASP items^a^ 25													Total
First author, year	1	2	3	4a	4b	4c	5	6	7	8	9	10	11	
Anderson‐Hanley et al., 2018^30^	Yes	Yes	Yes	No	No	No	Yes	Yes	Yes	No	Yes	Yes	Yes	9
Bahar‐Fuchs et al., 2017^31^	Yes	Yes	Yes	Yes	Yes	Yes	No	Yes	Yes	Yes	Yes	Yes	Yes	12
Hsieh et al., 2018^32^	Yes	Yes	Yes	No	No	No	Yes	Yes	Yes	No	Yes	Yes	Yes	9
Jelcic et al., 2014^33^	Yes	Yes	Yes	No	Yes	Yes	Yes	Yes	No	No	No	Yes	Yes	9
Karssemeijer 2019^11^, ^12^	Yes	Yes	Yes	No	No	No	Yes	Yes	Yes	Yes	Yes	Yes	Yes	10
Kwan et al., 2020^34^	Yes	Yes	Yes	No	No	No	Yes	Yes	Yes	No	No	Yes	Yes	8
Laver et al., 2020^35^	Yes	Yes	Yes	No	No	Yes	No	Yes	No	Yes	Yes	Yes	Yes	9
Li et al., 2021^36^	Yes	Yes	Yes	No	Yes	Yes	Yes	Yes	No	Yes	Yes	Yes	Yes	11
Oliveira et al., 2021^37^	Yes	Yes	Yes	No	No	No	Yes	Yes	Yes	No	Yes	Yes	Yes	9
Padala et al., 2012^38^	Yes	Yes	Yes	No	No	No	Yes	Yes	No	No	Yes	Yes	Yes	8
Padala et al., 2017^39^	Yes	Yes	Yes	No	No	No	Yes	Yes	No	No	Yes	Yes	Yes	8
Petersen et al., 2020^40^	Yes	Yes	Yes	No	No	Yes	No	Yes	No	No	Yes	Yes	Yes	8
Robert et al., 2021^41^	Yes	Yes	Yes	No	No	No	Yes	Yes	No	Yes	Yes	Yes	Yes	9
Schwenk et al., 2016^42^	Yes	Yes	Yes	No	No	No	Yes	Yes	Yes	No	Yes	Yes	Yes	9
Swinnen et al., 2021^43^	Yes	Yes	Yes	No	No	Yes	Yes	Yes	Yes	No	Yes	Yes	Yes	10
Tchalla et al., 2013^44^	Yes	Yes	Yes	No	No	No	Yes	Yes	Yes	Yes	Yes	Yes	Yes	10
van Santen et al., 2020^45^	Yes	Yes	Yes	No	No	No	Yes	Yes	Yes	Yes	Yes	Yes	Yes	10
Wiloth et al., 2018^46^	Yes	Yes	Yes	Yes	Yes	No	Yes	Yes	Yes	No	Yes	Yes	Yes	11
Yu et al., 2015^47^	Yes	Yes	Yes	No	Yes	Yes	Yes	Yes	No	No	Yes	Yes	Yes	10

^a^ Item 1: Did the study address a clearly focused research question? Item 2: Was the assignment of participants to interventions randomised? Item 3: Were all participants who entered the study accounted for at its conclusion? Item 4a: Were the participants ‘blind’ to intervention they were given? Item 4b: Were the investigators ‘blind’ to the intervention they were giving to participants? Item 4c: Were the people assessing/analysing outcome/s ‘blinded’? Item 5: Were the study groups similar at the start of the randomised controlled trial? Item 6: Apart from the experimental intervention, did each study group receive the same level of care (that is, were they treated equally)? Item 7: Were the effects of intervention reported comprehensively? Item 8: Was the precision of the estimate of the intervention or treatment effect reported? Item 9: Do the benefits of the experimental intervention outweigh the harms and costs? Item 10: Can the results be applied to your local population/in your context? Item 11: Would the experimental intervention provide greater value to the people in your care than any of the existing interventions?

### 3.2 Study characteristics

The studies (Table 2) were conducted in 13 countries, the largest number in the United States of America (*n* = 5; 26%). Most studies were RCTs (*n* = 8; 42%) and pilot RCTs (*n* = 9; 47%). Sixty‐eight percent (*n* = 13) included participants living with dementia and 32% (*n* = 6) participants with MCI. The studies had a mean sample of 57 participants (range: 17–112). The overall sample of this review included 1074 participants (mean age = 80 years).

**TABLE 2:** Study characteristics and findings

Author, year	Country	Design	Population	*n*	Mean age (SD)	Results as reported in studies. Effect sizes reported, if included in study reports	Conclusion
Anderson‐Hanley et al., 2018^30^	USA	RCT	MCI community dwellers	111	78.1 (9.9)	Three months versus baseline (within group comparisons, intervention group): Executive function: Exer‐tour: *d* = 0.49; *p* = 0.08 Exer‐score: *d* = 0.14; *p* > 0.05 Game only (control): *d* = 0.13; *p* > 0.05 Pedal only (control): *d* = 0.35; *p* = 0.01 Six months versus baseline (within group comparisons, intervention group): Executive function: Exer‐tour: *d* = 0.51; *p* = 0.049 Exer‐score: *d* = 0.47; *p* = 0.001 Verbal Memory: Exer‐tour: *p* = 0.003 Exer‐score: *p* = 0.047	After 6 months, exer‐tour and exer‐score yielded significant moderate effects on executive function. Both exer‐tour and exer‐score resulted in significant improvements in verbal memory
Bahar‐Fuchs et al., 2017^31^	Australia	RCT	MCI (with or without mood‐related neuro‐psychiatric symptoms)	43	76.0 (6.3)	Twelve weeks versus baseline (intervention vs. control): Global cognitive ability: *d* = 0.80; *p* < 0.01 Delayed memory: *d* = 0.25; *p* < 0.01 Learning and memory: *d* = 0.50; *p* < 0.01 Memory‐contentment: *d* = −0.27; *p* < 0.01 Memory‐mistakes: *d* = 0.40; *p* > 0.01 Memory‐Strategies: *d* = 0.00; *p* > 0.01 Memory Functioning Discrepancy: *d* = −0.09; *p* > 0.01 Composite mood: *d* = −0.07; *p* > 0.01 GDS: *d* = 0.09; *p* > 0.01 GAI: *d* = −0.14; *p* > 0.01 AES: *d* = −0.53; *p* < 0.01 Follow‐up (3 months after intervention) versus baseline (intervention vs. control): Global cognitive ability: *d* = 0.79; *p* < 0.01 Delayed memory: *d* = 0.92; *p* < 0.01 Learning and memory: *d* = 0.83; *p* < 0.01 Memory‐contentment: *d* = −0.37; *p* > 0.01 Memory‐mistakes: *d* = 0.46; *p* > 0.01 Memory‐Strategies: *d* = −0.06; *p* > 0.01 Memory Functioning Discrepancy: *d* = −0.23; *p* > 0.01 Composite mood: *d* = 0.15; *p* > 0.01 GDS: *d* = 0.10; *p* > 0.01 GAI: *d* = 0.27; *p* > 0.01 AES: *d* = 0.46; *p* > 0.01	There are cognitive benefits associated with a home‐based, tailored and adaptive Computerised Cognitive Training for older adults with cognitive impairment (with or without mood‐related neuropsychiatric symptoms) over and beyond the benefits of a non‐adaptive/non‐tailored active control training condition
Hsieh et al., 2018^32^	Taiwan	Quasi‐randomised clinical trial	Older adults with cognitive impairment	60	78.2 (7.7)	Three‐months versus baseline (Intervention vs. control) 6‐min walk test: *d* = 0.30; *p* = 0.16 30‐s sit‐to‐stand test: *d* = 0.69; *p* = 0.01 30‐s arm curl test: *d* = 20; *p* = 0.39 TUGT: *d* = 0.08; *p* = 0.74 Functional reach: *d* = 0.50; *p* = 0.04 Sit and reach: *d* = −0.03; *p* = 0.98 Drop ruler test: *d* = −0.26; *p* = 0.18 5‐m gait speed: *d* = −0.60^b^; *p* = 0.009 LTM: *d* = 0.04; *p* = 0.79 STM: *d* = −0.12; *p* = 0.55 ATTEN: *d* = 0.21; *p* = 0.43 MENMA: *d* = −0.53; 0.71 ORIEN: *d* = −0.17; 0.47 ABSTR: *d* = 0.74; *p* = 0.002 LANG: *d* = 0.22; *p* = 0.55 DRAW: *d* = 0.03; *p* = 0.95 ANML: *d* = −0.10; *p* 0.69 CASI: *d* = 0.14; *p* = 0.57 Six months versus baseline (Intervention vs. control): 6‐min walk test: *d* = 0.55; *p* = 0.001 30‐s sit‐to‐stand test: *d* = 0.82; *p* = 0.002 30‐s arm curl test: *d* = 0.16; *p* = 0.41 TUGT: *d* = −0.03; *p* = 0.81 Functional reach: *d* = 1.01; *p* = 0.00 Sit and reach: *d* = −0.22; *p* = 0.51 Drop ruler test: *d* = −0.28; *p* = 0.13 5‐min gait speed: *d* = −0.60; *p* = 0.009 LTM: *d* = 0.28; *p* = 0.26 STM: *d* = −0.53; *p* = 0.06 ATTEN: *d* = 0.42; *p* = 0.13 MENMA: *d* = −0.06; 0.86 ORIEN: *d* = −0.35; 0.10 ABSTR: *d* = 0.74; *p* = 0.002 LANG: *d* = 0.12; *p* = 0.58 DRAW: *d* = 0.27; *p* = 0.37 ANML: *d* = 0.04; *p* = 0.89 CASI: *d* = 0.26; *p* = 0.16	The VRTC exercise posed a protective effect for some cognitive and physical functions in older adults with CI
Jelcic et al., 2014^33^	Italy	Pilot RCT	Older adults with early Alzheimer's Disease living in elderly care facility	27	86 (5.1)	Three months versus baseline (Three‐group comparison: Lexical‐semantic stimulation through telecommunication technology vs. Lexical‐semantic stimulation in‐person vs. unstructured cognitive treatment): MMSE score: *p* = 0.03 Language, verbal naming: *p* = 0.003 Language, phonemic fluency: *p* = 0.04 Language, semantic fluency: *p* = 0.6 Verbal episodic memory, Story immediate recall: *p* = 0.01 Verbal episodic memory, Story delayed recall: *p* = 0.12 Verbal episodic memory, RAVL Immediate recall: *p* = 0.2 Verbal episodic memory, RAVL Delayed recall: *p* = 0.03	Clinical application of telecommunication technology to cognitive rehabilitation of elderly patients with neurodegenerative cognitive impairment may improve cognitive performance
Karssemeijer et al., 2019^11^, ^12^	Netherlands	RCT	Older people living with dementia	115	79.2 (6.9)	Twelve weeks versus baseline (Three‐group comparison: Exergame vs. aerobic vs. control group): EFIP score: *p* = 0.43 Ten‐Meter Walk Test, m/s: *p* = 0.11 TUGT: *p* = 0.07 Five‐time sit to stand test: *p* = 0.24 FICSIT‐4 score: *p* = 0.33 SPPB score: *p* = 0.18 PASE Score: *p* = 0.18 Katz Index: *p* = 0.12 Executive function: *p* = 0.338 Psychomotor speed: *p* = 0.004 Episodic memory: *p* = 0.184 Working memory: *p* = 0.153 Twelve weeks versus baseline (Two‐group comparison: Exergame vs. control group): Frailty index: *η* ^2^ = 0.05; *p* = 0.012 EFIP physical domain sub‐scale: *η* ^2^ = 0.087; *p* = 0.008 TUG: *η* ^2^ = 0.042; *p* = 0.07 Psychomotor speed: *η* ^2^ = 0.102; *p* = 0.009 Twenty‐four weeks versus baseline (Three‐group comparison: Exergame vs. aerobic vs. control group): Ten‐Meter Walk Test, m/s: *p* = 0.32 TUGT: *p* = 0.40 Five‐time sit to stand test: *p* = 0.23 FICSIT‐4 score: *p* = 0.37 SPPB score: *p* = 0.17 PASE Score: *p* = 0.26 Twenty‐four weeks versus twelve weeks (Three‐group comparison: Exergame vs. aerobic vs. control group): Executive function: *p* = 0.77 Psychomotor speed: *p* = 0.003 Episodic memory: *p* = 0.122 Working memory: *p* = 0.056	A 12‐week exergame intervention reduces the level of frailty in people with dementia
Kwan et al., 2020^34^	Hong Kong	Pilot RCT	Older people living with CI	33	71 (−)	Twelve weeks versus baseline (within group comparisons, intervention group): MoCA: *d* = 0.7; *p* = 0.003 FFI: *d* = −1.41; *p* = 0.007 PASE: *d* = 1.13; *p* = 0.002 Hand‐grip strength: *d* = 0.66; *p* = 0.009 Walking speed: *d* = −1.32; *p* = 0.001 Walking time: *d* = 0.23; *p* = 0.03 Step count: *d* = 0.39; *p* = 0.02 Brisk walking time: *d* = 0.58; *p* = 0.009 Peak cadence: *d* = 0.72; *p* = 0.003 Moderate‐to‐vigorous physical activity: *d* = 0.35; *p* = 0.04	A brisk walking intervention and behaviour change through mHealth can increase moderate‐to‐vigorous physical activity time to an extent sufficient to yield reduction in cognitive frailty in older people living with CI
Laver et al., 2020^35^	Australia	RCT	Older people living with dementia living in the community	63	79.4 (6.5)	Sixteen weeks versus baseline (within group comparison, intervention group): CAFU: *p* = 0.01 CAFU—Instrumental ADL: *p* = 0.001 CAFU—Basic ADL: *p* = 0.4482 Behavioural symptoms: *p* < 0.0001 Upset: *p* = 0.1674 Sixteen weeks versus baseline (Intervention vs. control): CAFU: *p* = 0.11 CAFU—Instrumental ADL: *p* = 0.11 CAFU—Basic ADL: *p* = 0.46 Behavioural symptoms: *p* = 0.0003 Upset: *p* = 0.98	It is feasible to offer dyadic interventions via telehealth and doing so reduces travel time and results in similar benefits for families than face‐to‐face delivery
Li et al., 2021^36^	USA	Feasibility RCT	Older adults with MCI living in the community	30	76.1(6.2)	Twenty‐four weeks versus baseline (intervention vs. control): Falls: *p* = 0.07 Injurious falls: *p* = 0.57 4‐Stage Balance Test: *p* = 0.02 30‐s chair stands: *p* < 0.001 TUGT: *p* < 0.001	Findings from this study suggest the potential efficacy of implementing an at‐home, virtual, interactive Tai Ji Quan program, delivered in real‐time, as a potential balance training and falls prevention intervention for older adults with MCI
Oliveira et al., 2021^37^	Portugal	Pilot RCT	People living with mild to moderate dementia in a residential care home	17	83.2 (5.6)	Two months versus baseline (within group comparison—Intervention group): FAB: *η* ^2^ = 0.119; *p* = 0.174 MMSE: *η* ^2^ = 0.24; *p* = 0.033 TMT part A: *η* ^2^ = 0.44; *p* = 0.063 TMT part B: *η* ^2^ = 0.26; *p* = 0.063 IADL: *η* ^2^ = 0.001; *p* = 0.905 GSD: *η* ^2^ = 0.35; *p* = 0.058 CDR: *p* > 0.05 Two months versus baseline (intervention vs. control): MMSE: *p* = 0.056 CDT: *p* > 0.05	Virtual Reality‐Based Cognitive Stimulation is effective for maintaining cognitive function in people living with dementia
Padala et al., 2012^38^	USA	Pilot RCT	People living with mild dementia in an assisted facility	22	79.3(9.8)	Eight weeks versus baseline (within group comparison—Intervention group): BBS: *p* = 0.003 Tinetti Score: *p* = 0.013 TUG: *p* = 0.31 ADL: *p* = 0.55 IADL: *p* = 0.36 MMSE: *p* = 0.93 Eight weeks versus baseline (intervention vs. control): BBS: *p* = 0.56 Tinetti Score: *p* = 0.97 TUG: *p* = 0.52 ADL: *p* = 0.11 IADL: *p* = 0.11 MMSE: *p* = 0.70	Use of Wii‐Fit resulted in significant improvements in balance and gait in people living with mild dementia in an assisted facility
Padala et al., 2017^39^	USA	Pilot RCT	Older adults with mild dementia living in the community	30	73 (6.2)	Eight weeks versus baseline (Intervention vs. control): BBS: *p* < 0.001 ABC: *p* < 0.001 FES: *p* = 0.002 MMSE: *p* = 0.0264 ADL: *p* = 0.708 IADL: *p* = 0.316 Sixteen weeks versus baseline (Intervention vs. control): BBS: *p* < 0.001 ABC: *p* = 0.182 FES: *p* = 0.129 MMSE: *p* = 0.147 ADL: *p* = 0.449 IADL: *p* = 0.267	Home‐based, caregiver‐supervised Wii‐Fit exercises improve balance and may reduce fear of falling in community‐dwelling older adults with mild dementia
Petersen et al., 2020^40^	Denmark	Pilot RCT	Older adults with dementia living at home	26	75.6 (6.4)	15 weeks versus baseline (within group comparison—Intervention group): Sit to stand: *p* > 0.05 10MDW: *p* < 0.05 TUG: *p* > 0.05 6‐min walking test: *p* > 0.05 MMSE: *p* > 0.05 NPI: *p* > 0.05 27 weeks versus baseline (Intervention vs. control) Sit to stand: *p* > 0.05 10MDW: *p* > 0.05 TUG: *p* > 0.05 6‐min walking test: *p* > 0.05 MMSE: *p* > 0.05 NPI: *p* > 0.05	Physical function tended to remain stable or even improved among people living with dementia following a home‐based virtual reality physical training intervention
Robert et al., 2021^41^	France	RCT	Older people living with mild or major neurocognitive disorder in the community	91	81.7(7.9)	Twelve weeks versus baseline (within group comparison—Intervention group): NPI: *p* = 0.63 Twenty‐four weeks versus baseline (within group comparison—Intervention group): MMSE: *p* = 0.748 NPI: *p* = 0.001 AI: *p* = 0.388 Twenty‐four weeks versus baseline (intervention vs. control): MMSE: *p* = 0.557 NPI: *p* = 0.008 AI: *p* = 0.032	The use of exergame combining motor and cognitive activities improved apathy in older people living with mild or major neurocognitive disorder
Schwenk et al., 2016^42^	USA	Pilot RCT	Community dwelling older people living with MCI	22	78.2(8.7)	Four weeks versus baseline (intervention vs. control): Fear of falling: *η* ^2^ = 0.302; *p* = 0.015 Balance, eyes open, centre of mass, sway (area): *η* ^2^ = 0.22; *p* = 0.04 Balance, eyes open, centre of mass, sway (mediolateral): *η* ^2^ = 0.21; *p* = 0.04 Balance, eyes open, centre of mass, sway (anterior posterior): *η* ^2^ = 0.25; *p* = 0.03 Balance, eyes closed, centre of mass, sway (area): *η* ^2^ = 0.07; *p* = 0.27 Balance, eyes closed, centre of mass, sway (mediolateral): *η* ^2^ = 0.10; *p* = 0.19 Balance, eyes closed, centre of mass, sway (anterior posterior): *η* ^2^ = 0.11; *p* = 0.18 Gait—Habitual walking (speed): *η* ^2^ = 0.05; *p* = 0.35 Gait—Habitual walking (stride time variability): *η* ^2^ = 0.005; *p* = 0.78 Gait—Fast walking (speed): *η* ^2^ = 0.09; *p* = 0.22 Gait—Fat walking (stride time variability): *η* ^2^ = 0.03; *p* = 0.83 FESI: *η* ^2^ = 0.30; *p* = 0.01 MoCA: *η* ^2^ = 0.12; *p* = 0.13 Trail A: *η* ^2^ = 0.09; *p* = 0.68 Trail B: *η* ^2^ = 0.006; *p* = 0.74	Results suggest that sensor‐based training is beneficial for improving postural control in community dwelling older people living with MCI
Swinnen et al., 2021^43^	Switzerland	Pilot RCT	Older people living with major neurocognitive disorder residing in long‐term care facilities	45	85 (6.0)	Eight weeks versus baseline (Intervention vs. control): Gait speed: *η* ^2^ = 0.41; *p* < 0.001 SPPB: *η* ^2^ = 0.64; *p* < 0.001 Step reaction time test: *η* ^2^ = 0.51; *p* < 0.001 MoCA: *η* ^2^ = 0.38; *p* < 0.001 NPI: *η* ^2^ = 0.05; *p* = 0.16 CSDD: *η* ^2^ = 0.43; *p* < 0.001 ADL: *η* ^2^ = 0.16; *p* = 0.008	An individually adapted exergame training improves lower extremity functioning, cognitive functioning and step reaction time and symptoms of depression in people living with major neurocognitive disorder residing in long‐term care facilities
Tchalla et al., 2013^44^	France	Pilot RCT	Older people living with dementia living at home	96	86.6 (6.5)	Intervention versus control Risk of fall: OR = 0.37; *p* = 0.024	The use of Home‐based technology coupled with teleassistance service significantly reduced the incidence of falls among elderly people living with mild‐to‐moderate dementia
van Santen et al., 2020^45^	Netherlands	RCT	Older people living with dementia living in the community	112	79.0 (6.0)	Three months versus baseline (intervention vs. control): SPPB: *d* = 0.14; *p* = 0.47 Physical activities per week: *d* = 0.30; *p* = 0.18 MMSE: *d* = 0.09; *p* = 0.5 TMT—Part A: *d* = −0.12; *p* = 0.48 TMT—Part B: *d* = 0.23; *p* = 0.40 IMI01: *d* = −0.02; *p* = 0.62 IMI02: *d* = 0.02; *p* = 0.66 IMI03: *d* = −0.04; *p* = 0.44 IMI04: *d* = 0.03; *p* = 0.50 IMI05: *d* = 0.00; *p* = 0.96 Psychological wellbeing: *d* = 0.01; *p* = 0.7 PASE: *d* = −0.02; *p* = 0.68 GIP: *d* = 0.01; *p* = 0.90 Number of falls: *d* = −0.20; *p* = 0.26 Six months versus baseline (intervention vs. control): SPPB: *d* = 0.11; *p* = 0.73 Physical activities per week: *d* = 0.28; *p* = 0.12 MMSE: *d* = 0.36; *p* = 0.007 TMT—Part A: d = −0.37; p = 0.029 TMT—Part B: *d* = 0.00; *p* = 1.00 IMI01: *d* = −0.31; *p* = 0.13 IMI02: *d* = −0.21; *p* = 0.33 IMI03: *d* = −0.00; *p* = 0.99 IMI04: *d* = −0.36; *p* = 0.13 IMI05: *d* = 0.03; *p* = 0.91 Psychological wellbeing: *d* = 0.14; *p* = 0.47 PASE: *d* = −0.31; *p* = 0.09 GIP: *d* = −0.49; *p* = 0.03 Number of falls: *d* = −0.06; *p* = 0.92	Cycle exergaming yields some small to moderate positive effects on cognitive and social functioning in people living with dementia
Wiloth et al., 2018^46^	Germany	RCT	Older people living with dementia in the community	99	82.9 (5.8)	Ten weeks versus baseline (intervention vs. control): Physiomat^®^ Follow the ball task (accuracy): *η* ^2^ = 0.14 *p* < 0.001 Physiomat^®^ Follow the ball task (duration): *η* ^2^ = 0.25; *p* < 0.001 Physiomat^®^ Trail Making Score: *η* ^2^ = 0.211; *p* < 0.001 Three months versus baseline (intervention vs. control): Physiomat^®^ Follow the ball task (accuracy): *η* ^2^ = 0.25; *p* < 0.001 Physiomat^®^ Follow the ball task (duration): *η* ^2^ = 0.05; *p* = 0.04 Physiomat^®^ Trail Making Score: *η* ^2^ = 0.03; *p* = 0.14	Computer game‐based motor cognitive training has the potential to improve motor‐cognitive performances in people living with dementia in the community
Yu et al., 2015^47^	China	RCT	Older Chinese Adults with Mild‐to‐Moderate Dementia	32	83 (−)	Eight weeks versus baseline (within group comparison): MoCA language sub‐scores: *p* < 0.05 MoCA attention sub‐scores: *p* > 0.05 MoCA digit‐span sub‐scores: *p* > 0.05 MMSE total score: *p* > 0.05 CVFT: *p* > 0.05 BPSD: *p* < 0.05 NPI: *p* < 0.05 CMAI: *p* > 0.05 CSDD: *p* > 0.05 Eight weeks versus baseline (Intervention vs. control): CMAI: *p* < 0.05 CMAI—Verbally aggressive sub‐score: *p* < 0.05 CSDD: *p* > 0.05	Touch‐screen videogame training can alleviate behavioural symptoms in older adults with mild‐to‐moderate dementia and may improve cognitive functioning

Abbreviations: 10MDW, 10 Meter Dual task Walking test; ABC, Activities Specific Balance Scale; ABSTR, abstract thinking and judgment; ADL, Activities of Daily Living; AES, Apathy Evaluation Scale; AI, Apathy Inventory; ANML, animal name fluency; ATTEN, attention; BBS, Berg Balance Scale; BPSD, Behavioural Psychological Symptoms of Dementia; CAFU, Caregiver Assessment of Function and Upset; CASI, Cognitive Abilities Screening Instrument; CDT, Clock drawing test; CMAI, Cohen‐Mansfield Agitation Inventory; CSDD, Cornell Scale for Depression in Dementia; CVFT, category verbal fluency tests; *d*, Cohen's *d*; DRAW, drawing; FAB, Frontline Assessment Battery; FES, Falls Efficacy Scale; FFI, Fried Frailty Index; FICSIT‐4, Frailty and Injuries Cooperative Studies of Intervention Techniques Subtest; GAI, Geriatric Anxiety Scale; GDS, Geriatric Depression Scale; GIP, Behaviour Observation Scale for Intramural Psychogeriatrics: subscale 1 (unsocial behaviour); HR, Hazard Ratio; IADL: Instrumental activities of daily living; IMI01, Intrinsic Motivation Inventory, subscale 1 interest/enjoyment in physical exercise; IMI02, Intrinsic Motivation Inventory, subscale 2 perceived competence in physical exercise; IMI03, Intrinsic Motivation Inventory, subscale 3 effort in/importance of physical exercise; IMI04, Intrinsic Motivation Inventory, subscale 4 perceived choice of physical exercise; IMI05, Intrinsic Motivation Inventory, subscale 5 value/usefulness of physical exercise; LANG, language; LTM, long‐term memory; MMSE, Mini Mental State Examination; MoCA, Montreal Cognitive Assessment; MENMA, mental manipulation; NPI, Neuro Psychiatric Inventory; OR, Odds Ratio; ORIEN, orientation; PASE, Physical Activity Scale for the Elderly; RAVL, Rey Auditory Verbal Learning Test; RCT, Randomised Controlled Trial; SPPB, Short Physical Performance Battery; STM, short‐term memory; TMT, Trail Making test; TUGT, Timed Up and Go Test; *η* ^2^, Partial Eta Squared.

### 3.3 Types of interventions, design, content, and delivery features

Nineteen interventions were included in the studies (two studies reported the same intervention).^11^, ^12^ The interventions were diverse (Table 3), comprising one or more components. Exergaming (i.e., video games that are also a form of exercise), either including a physical element only or a combination of physical and cognitive elements, was the most common intervention (*n* = 7; 37%). Five interventions (26%) were virtual reality‐based (i.e., a computer that simulates the real world), three (16%) included videogaming (without a physical exercise element), two (11%) delivered telehealth (e.g., online consultations or rehabilitation), two (11%) used assistive technology (i.e., equipment to increase, maintain, or improve the functional capabilities), and one (5%) was an online class.

**TABLE 3:** Intervention characteristics—Adapted from the template for intervention description and replication (TIDieR) checklist and guide

Author, year	Type of intervention	Design (how)—the *modes of delivery*	Content (what)—the *materials, procedures, activities, and/or processes*	Delivery (who, where, when, how much)—*format of the intervention delivery, the location, duration of intervention, length of sessions, frequency of sessions, intensity*
Anderson‐Hanley et al.^30^	Exer‐tour (relatively cognitively passive) Exer‐score (cognitively effortful)	A virtual reality‐enhanced, recumbent stationary bike	Exer‐tour: Participants pedal along scenic bike paths; involves steering but cannot leave road or crash into anything. Exer‐score: Participants pedal in 360‐degree radius to locate coloured coins and matching coloured dragons of varying speed/difficulty	Format: Individual Location: sites in the community (e.g., retirement communities, YMCAs) Duration: 24 weeks Length: 45 min Frequency: 3/5 times/week Intensity: based on individual heart rate monitoring
Bahar‐Fuchs et al.^31^	Computerised Cognitive Training	A commercially available computerised cognitive training platform (Cognifit™) online	Participants engage with standardised, game‐like computer tasks. Psychoeducation, and a range of behaviour‐change techniques are used to optimise engagement, adherence, and perseverance	Format: Individual Location: participants' homes Duration: 8‐12 weeks Length: 20‐30 min Frequency: 3 times/week Intensity: Individually tailored and adaptive (i.e., level of difficulty continuously adapted on participant's performance, with successful completion of one level of difficulty resulting in an increased difficulty on the subsequent)
Hsieh et al.^32^	Virtual Reality‐based Tai‐Chi	Your Shape Fitness Evolved 2012 Zen energy classes on Xbox 360 Kinect	A Kinect sensor device captures one player's motion and provides feedback. On the screen, the player must follow the movements of a virtual coach. When the right motion is performed, the player on the screen becomes brighter. Other participants stand around the instructor and exercise together	Format: Group, instructor‐led Location: ‐ Duration: 24 weeks Length: 60 min Frequency: twice/week Intensity: eight activities, ranging in difficulty from easy to hard. Players need to pass them to unlock more advanced/difficult activities.
Jelcic et al.^33^	Lexical‐semantic stimulation through telecommunication technology (LSS‐tele) with in‐person LSS (LSS‐direct) and unstructured cognitive treatment (UCS)	Rehab exercises provided through personal computer workstations using Windows 7 or XP operating systems; teleconference through Skype	Lexical tasks aimed at enhancing semantic verbal processing delivered through remote control based on telecommunication technology. The exercises focused on the interpretation of written words, sentences, and stories	Format: Group, instructor‐led Location: elderly care home Duration: 12 weeks Length: 60 min Frequency: twice/week Intensity: ‐
Karssemeijer et al., 2019^11^, ^12^	Cognitive‐aerobic bicycle exergame	Stationary bike connected to a video screen	Participants pedal following a route through a familiar digital environment (e.g., a city) while performing cognitive tasks incorporated in the cycling routes that are shown on the video screen	Format: Individual Location: Community centre Duration: 12 weeks Length: 30‐50 min Frequency: 3 times/week Intensity: 65%–75% of heart rate reserve; different cognitive training levels, changing with user's performance
Kwan et al., 2020^34^	Brisk Walking Intervention and behaviour change through mHealth	Samsung Galaxy smartphone J2 with 2 apps (i.e., Samsung Health and WhatsApp)	Participants set weekly goals of brisk walking. Participants wear a step‐counter during week. Participants receive WhatsApp weekly routine messages, messages when there is no brisk walking for more than 2 days, and praise message when the weekly goal is achieved earlier than expected	Format: Individual Location: Anywhere the participant walks Duration: 12 weeks Length: 60 min Frequency: 7 times/week Intensity: Based on baseline fitness and progress
Laver et al., 2020^35^	Telehealth delivery of a dyadic dementia care intervention	Personal device (laptop, tablet, or smartphone) or tablet on loan with videoconferencing software (Cisco Webex)	Participants, caregivers and environment are assessed by OT OT works with caregiver to problem solve, educate, build skills, and enhance activity engagement in the person with dementia	Format: Individual, delivered by OT Location: Participant's home Duration: 16 weeks Length: 60 min Frequency: once/fortnight Intensity: Tailored to the capabilities and interests of the participant, caregiver and environment
Li et al., 2021^36^	Online virtual falls prevention intervention through a dual‐task Tai Ji Quan training program	iPad or smartphone with Zoom App	Participants receive 10–15 min of preparatory exercises, 45–50 min of core training (learning, practicing) and 1–2 min of closing exercises. Within a dual‐task framework, the training also involves concurrent cognitive exercises aimed at challenging multiple cognitive domains (memory, executive function, spatial orientation, and processing speed)	Format: Group, instructor‐led Location: Participant's home Duration: 24 weeks Length: 60 min Frequency: once/week Intensity: ‐
Oliveira et al., 2021^37^	Virtual Reality‐Based Cognitive Stimulation	Computer with non‐immersive VR exposure on a laptop screen of 17 inches	The participant undertakes activities inside a virtual apartment relating to morning hygiene, shoe closet test, wardrobe test, memory test, virtual kitchen, TV News. The participant also undertakes outdoor tasks, navigating to each of the locations in a virtual city, including grocery store, pharmacy, and art gallery	Format: Individual, clinical neuropsychologist‐delivered Location: Residential care home Duration: 8 weeks Length: 45 min Frequency: twice/week Intensity: different difficulty levels for progression throughout the intervention
Padala et al., 2012^38^	Strength, yoga, and balance exergaming	Nintendo Wii‐Fit console connected to a mobile television unit	The participant spends 10 min doing yoga, 10 min doing strength training, and 10 min doing balance games	Format: individual, researcher‐supervised Location: exercise room of a residential care home Duration: 8 weeks Length: 30 min Frequency: 5 times/week Intensity: ‐
Padala et al., 2017^39^	Interactive video‐game‐led physical exercise program	Nintendo Wii‐Fit console connected to a television unit	The participant performs exercises of yoga, strength training, aerobics, balance games, and training plus, which includes more complex exercise tasks. Each session includes a warm‐up, exercise, and cool down phase	Format: individual, caregiver‐supervised Location: Participant's home Duration: 8 weeks Length: 30 min Frequency: 5 times/week Intensity: Starts at level one, subsequent levels are opened automatically upon completion of previous levels
Petersen et al., 2020^40^	Virtual reality physical training plus group face‐to‐face training	The virtual reality hardware consists of a touchscreen, a Microsoft Kinect camera, and a modem^a^	The participant is guided through exercises via text, recorded instructions, and animations The Kinect camera detects movements and corrects possible errors with onscreen feedback; once the participant successfully completes each exercise, visual feedback in the form of a green smiling icon is displayed onscreen and level can be advanced^a^	Format: individual Location: Participant's home Duration: 12 weeks Length: 20 min Frequency: twice/week Intensity: Starts at level one, subsequent levels are opened automatically upon completion of previous levels^a^
Robert et al., 2021^41^	Exergame combining motor and cognitive activities	The X‐Torp exergame is played on a desktop PC and displayed on a high‐resolution wide screen. Participant interacts with the exergame using a Red Green Blue + Depth Kinect	The participant can: 1. play in scenario mode action game dynamics (moving a submarine); 2. Explore open environments (reaching islands) where access is granted through playful mini‐games and orientation exercises	Format: individual or group, therapist‐controlled Location: memory centres, day care centres, and nursing homes Duration: 12 weeks Length: 15 min Frequency: twice/week Intensity: therapist can modify/adjust the game difficulty, based on participant's performance
Schwenk et al., 2016^42^	Sensor‐based balance training programme	A 24‐inch computer screen, an interactive virtual user interface, and five inertial sensors	The participant does ankle point‐to‐point reaching tasks and virtual obstacle crossing tasks. Live feedback is provided	Format: individual, supervised Location: memory clinic Duration: 4 weeks Length: 45 min Frequency: twice/week Intensity: progressive
Swinnen et al., 2021^43^	Stepping exergame	The exergame device “Dividat Senso”, consisting of a step training platform which is sensitive to pressure changes, connected via a USB cable to a computer and a frontal television screen on which the exergames are displayed	The participant plays multiple games lasting 120–200 s. Starting from an upright stance with both feet in the middle of the platform, the participant interacts with the game interface by pushing one foot on one of the four different arrows. The device provided real‐time visual, auditory and somatosensory (vibrating platform) cues, and feedback	Format: individual, supervised Location: care home Duration: 8 weeks Length: 15 min Frequency: 3 times/week Intensity: automatically adapted, providing more difficult stimuli when the players reacted fast and correct
Tchalla et al., 2013^44^	Home‐based technology coupled with teleassistance service	The home‐based technology consists of a nightlight path Teleassistance service includes a remote intercom, an electronic bracelet and a central hotline providing telephone support	The participant activates a wire sensor installed on the floor near the bed when getting up that turns on a nightlight path. The participant can ask for help if they fall by using the remote intercom, the electronic bracelet. A central hotline providing telephone support will help	Format: individual Location: participant's home Duration: ‐ Length: ‐ Frequency: ‐ Intensity: ‐
van Santen et al., 2020^45^	Exergaming combining physical exercise (interactive cycling) with cognitive stimulation	Stationary bicycle connected to a screen	While cycling, the Participant sees a route on the screen. They can pick a route, and it mimics the experience of cycling outside, thus offering simultaneous physical and cognitive stimulation	Format: individual Location: day care centre Duration: 24 weeks Length: ‐ Frequency: twice/week Intensity: ‐
Wiloth et al., 2018^46^	Computer game‐based motor cognitive training	Physiomat®, a pressure‐sensitive step training platform	The participant moves a cursor from the centre of the screen directly to the targets highlighted as a moving yellow ball on the screen as fast as possible by shifting their weight while holding onto the handles of Physiomat®. As difficulty progresses, the participant is asked to move the cursor on the screen in order to connect an increasing number of digits	Format: group, supervised Location: research centre Duration: 10 weeks Length: 90 min Frequency: twice/week Intensity: increasing, based on performance
Yu et al., 2015^47^	Computer‐assisted Intervention using Touch‐screen Video Game Technology	Interactive touch screens/displays (Sur 40, I‐pad, optical touch computer screen)	The participant plays four touch‐screen video games, including (1) Bingo (provided a figure, identify the same figure in a table with different figures), (2) Connect the dot ultimate (connect the dots by pressing the number on the dots in an ascending order to draw a cartoon figure), (3) Find difference (find the differences between two photos by pressing the point of difference within a time limit), (4) Mosquito splash (press the mosquitoes on the screen, but avoid butterflies)	Format: individual, researcher‐supervised Location: Geriatric day hospital Duration: up to 8 weeks Length: 30 min Frequency: once‐twice/week Intensity: ‐

*Note*: Meta‐analyses of the effects (positive and negative) of the interventions on physical, cognitive, behavioural and psychological outcomes, and ADLs.

^a^ Information refers to the exergaming component only.

In relation to delivery features (i.e., who, where, when, how much), 14 (74%) interventions were delivered individually and five (26%) in a group. Twelve interventions (63%) were supervised, in seven (37%) the participants were unassisted. Seven interventions (37%) were delivered in the participants' homes, five (26%) in care/nursing homes, four (21%) in clinical community settings (e.g., hospitals), one (10%) in non‐clinical community settings (e.g., community centres), and one (5%) in research facilities. The average duration of the interventions was 13 weeks (range: 4–24 weeks) and the average length of each session was 43 min (range: 15–90 min). Participants were asked to have sessions three times/week on average (range: once/fortnight–once/day). Most interventions' intensity was adapted on participants' performance (*n* = 9; 47%; e.g., completion of one level unlocked a new more difficult level), heart monitoring (*n* = 2; 11%), and individual needs (*n* = 1; 5%).

**FIGURE 2:**
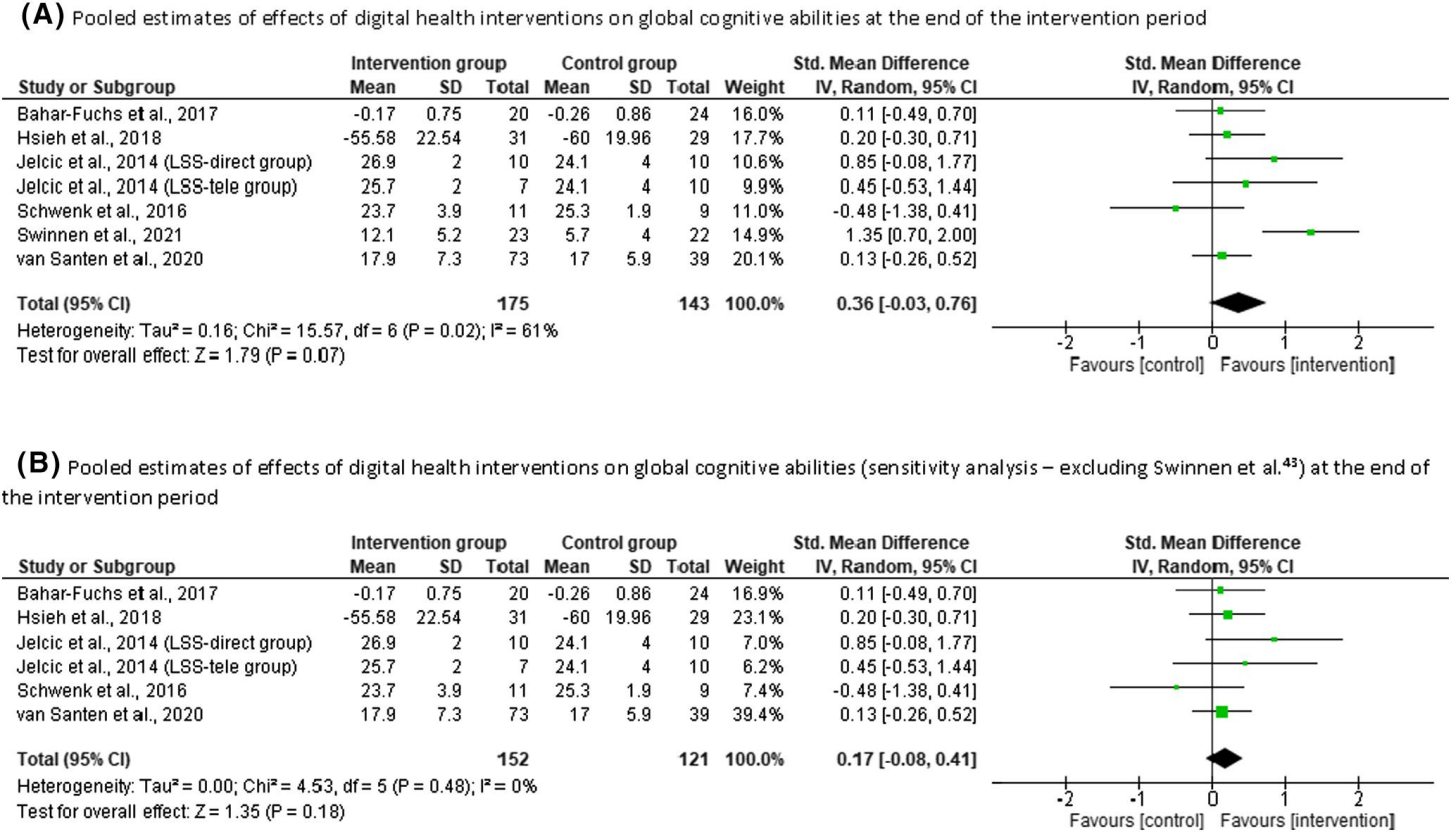
(A) Pooled estimates of effects of digital health interventions on global cognitive abilities at the end of the intervention period. (B) Pooled estimates of effects of digital health interventions on global cognitive abilities (sensitivity analysis—excluding Swinnen et al.^43^) at the end of the intervention period

**FIGURE 3:**
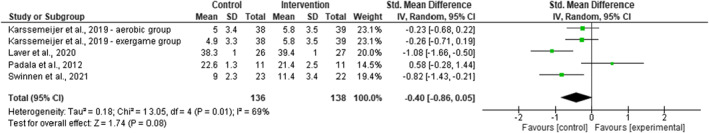
Pooled estimates of effects of digital health interventions on basic ADLs at the end of the intervention period

Meta‐analyses were only feasible with two outcomes: overall cognitive abilities and basic ADLs. Based on evidence from six studies (Jelcic et al.^33^ had two intervention groups; *n* = 318), we found that the digital health interventions produced a moderate improvement in overall cognitive abilities of participants with MCI/dementia (SMD = 0.36; 95% CI = −0.03 to 0.76; *I* ^2^ = 61%), compared to control conditions (Figure 2A). The sensitivity analyses found that only one study^43^ substantially affected heterogeneity. When this study was excluded from the pooled data, the aggregated treatment effect of interventions was small (SMD = 0.17; 95% CI = −0.08, 0.41; *I* ^2^ = 0%; Figure 2B). Based on evidence from five intervention groups (Karssemeijer et al.^11^, ^12^ had two intervention groups; *n* = 274), we found that the digital health interventions produced a negative moderate effect on basic ADLs of participants with MCI/dementia (SMD = −0.40; 95% CI = −0.86 to 0.05; *I* ^2^ = 69%), compared to the control conditions (Figure 3). The sensitivity analyses found that no study substantially affected heterogeneity.

### 3.4 *Positive effects* on outcome parameters

All results are in Table 1. In the next section, only a summary of results will be reported.

### 3.5 Physical outcomes

Physical outcomes were reported in 12 studies (60%), and included functional outcomes, motor‐cognitive performance, frailty, balance, risk of falls and dependence.

In terms of functional outcomes, Hsiesh et al.^32^ found that a group‐based, instructor‐led 6‐month virtual reality Tai‐Chi intervention yielded moderate to large improvements in people living with MCI (6‐min walk test: *d* = 0.55; *p* = 0.001; 30‐s sit‐to‐stand test: *d* = 0.82; *p* = 0.002; Functional reach: *d* = 1.01; *p* = 0.00; 5‐m gait speed: *d* = −0.60; *p* = 0.009). Significant improvements were found by Kwan et al.,^34^ comparing baseline and 12‐week measures in participants with MCI receiving a brisk walking intervention supplemented with behaviour change (Walking time: MD = 57.9 min/day; *p* = 0.03; Step count: MD = 3778.9; *p* = 0.02; Brisk walking time: MD = 3.1 min/day; *p* = 0.009; Peak cadence: MD = 7.0 steps/min; *p* = 0.003).

In relation to motor‐cognitive performance, dual task walking test scores were significantly improved in people with dementia in receipt of a 12‐week virtual reality physical training intervention,^40^ compared to baseline and to face‐to‐face delivery (10‐meter Dual‐Task Walking Test^48^: *p* < 0.05). Statistically significant reduction in frailty was reported by Karssemeijer et al.,^11^, ^12^ following a 3‐month cognitive‐aerobic bicycle exergame intervention delivered in community settings (Frailty index MD = −0.034; *p* = 0.012).

Improvements in balance measures were reported in participants with MCI^36^ receiving Tai Ji Quan training within a dual‐task framework, compared to stretching exercises only (4‐Stage Balance Test^49^ MD = 0.68; *p* = 0.02). Two RCTs^38^, ^39^ found that balance improved in participants living with dementia living in the community following an 8‐week supervised strength, yoga, and balance exergaming intervention (Berg Balance Scale^50^: *p* < 0.001); (Falls Efficacy Scale^51^: *p* = 0.002), as well as in people living with dementia in care homes (Berg Balance Scale^50^: *p* = 0.003); (Tinetti Score^52^: *p* = 0.013).

Risk of falls was significantly reduced in people with dementia after a home‐based intervention consisting of assistive technology (e.g., a nightlight path) and teleassistance service (i.e., a remote intercom, an electronic bracelet and a central hotline providing telephone support; OR = 0.37; *p* = 0.024).^44^ The only dyadic intervention included in this review generated improvements in dependence measures following 16 weeks of occupational therapy to problem solve, educate, build skills, and enhance activity in the person with dementia and caregiver, compared with baseline (Caregiver Assessment of Function and Upset—CAFU^53^: between difference = 6.0; *p* = 0.01) and the control condition (face‐to‐face; Caregiver Assessment of Function and Upset: 3.9; *p* = 0.11).^35^

### 3.6 Cognitive outcomes

Cognitive outcomes included executive function, memory, language, attention and global cognitive abilities, and were reported in eight studies (40%).

One study^30^ found that participants with MCI living in the community experienced significant positive effects on executive function (*d* = 0.47; *p* = 0.001) and verbal memory (*p* = 0.04) after 6 months of a virtual reality‐enhanced, recumbent stationary bike intervention with cognitive tasks.

In terms of language, Jelcic et al.^33^ found that 3‐month lexical‐semantic stimulation rehabilitation exercises provided through personal computers and teleconferencing in participants living with dementia in care homes yielded improvements in phonetic fluency score (18.1 vs. 14.3: *p* = 0.04) and semantic fluency score (20.4 vs. 17.9: *p* = 0.03), compared to baseline.

Two studies reported positive gains in attention. Jelcic et al.^33^ found a significant improvement in attention ability score in people with dementia living in care homes after a group, instructor‐led cognitive stimulation intervention, compared to baseline (38.0 vs. 35.6; *p* = 0.01). van Santen et al.^45^ reported improvements in the same population, following a 6‐month exergaming intervention combining interactive cycling with cognitive stimulation (Trail Making Test Part A^54^: *d* = 0.37; *p* = 0.029).

In terms of global cognitive abilities, Bahar‐Fuchs et al.^31^ found that an intervention providing game‐like computer tasks accompanied by therapist‐delivered behaviour‐change techniques to participants with MCI improved global cognition (*d* = 0.80; *p* < 0.01). Promising results were also found in a population living with dementia. An RCT evaluating the effectiveness of lexical‐semantic computer exercises^33^ found an increase in Mini Mental State Examination (MMSE)^55^ scores when comparing 3 months and baseline (25.7 vs. 23.7; *p* = 0.03).

### 3.7 Behavioural and psychological outcomes

Behavioural and psychological outcomes included depression, apathy, non‐social behaviour, agitation/verbally aggressive behaviour, and confidence/fear of falling, and were reported in eight studies (40%).

Swinnen et al.^43^ found a reduction in depressive symptoms among participants living with dementia living in care homes as a result of participation in a supervised stepping exergame intervention, compared to the control condition (listening to music), over an 8‐week period (*η* ^2^ = 0.43; *p* < 0.001). One study investigated apathy^41^ and found that an exergame intervention combining motor and cognitive activities delivered in the community generated a reduction in apathy in people living with dementia (Apathy Inventory)^56^ (*p* = 0.044).

In terms of behavioural symptoms, positive outcomes were found in an RCT evaluating a computer‐assisted intervention delivering touch‐screen cognitive videogames, in which participants living with dementia experienced a reduction in agitation (ES = 0.84; *p* < 0.05) and verbally aggressive behaviour (ES = 0.84; *p* < 0.05).^47^

A number of studies investigated changes in confidence. Bahar‐Fuchs et al.^31^ found that participants with MCI reported being more confident about their own memory following home‐based computerised cognitive training (t = 3.0, *p* < 0.01). Padala et al.^39^ recorded improvements in balance confidence in participants living with dementia receiving an 8‐week strength, yoga, and balance exergaming intervention (*p* < 0.001).

### 3.8 ADLs

ADLs were investigated in five studies (25%). Laver et al.^35^ found that an occupational therapy intervention delivered through telehealth produced benefits for the participants living with dementia in instrumental ADLs (*p* = 0.11) and basic (*p* = 0.46) ADLs. Padala et al.^38^, ^39^ found that an exergame intervention based on strength, yoga, and balance exercises also improved Instrumental ADL (*p* = 0.11) and ADLs (*p* = 0.11) in people with dementia living in the community and assisted facilities. Swinnen et al.^43^ reported a statistically significant improvement in ADLs (*p* = 0.008) among participants with major neurocognitive disorder residing in long‐term care facilities following a stepping exergaming intervention.

### 3.9 Interventions linked to *largest improvements* on outcome parameters

In relation to physical outcomes, largest effect sizes on lower limb function were reported by Hsiesh et al.^32^ (*d* = 0.82) and Swinnen et al.^43^ (*η* ^2^ = 0.41–0.64; *d* > 0.80). While Schwenk et al.^42^ also found large improvements in balance (*η* ^2^ = 0.26; *d* = 0.80), the largest effect size on balance was reported in Hsieh et al.^32^ (*d* = 1.01). Kwan et al.^34^ reported a large reduction in frailty (*d* = −1.41), Swinnen et al.^43^ on step reaction time (*η* ^2^ = 0.51; *d* > 0.80), and Wiloth et al.^46^ in motor‐cognitive performance (*η* ^2^ = 0.21; *d* > 0.80).

In relation to cognitive outcomes, while both Bahar‐Fuchs et al.^31^ (*d* = 0.80) and Oliveira et al.^37^ (*η* ^2^ = 0.24; *d* > 0.80) reported large effect sizes on global cognitive ability, the largest effect size for this outcome was found in Swinnen et al.^43^ (*η* ^2^ = 0.38; *d* > 0.80). Regarding behavioural and psychological outcomes, Swinnen et al.^43^ found large reduction in depression score (*η* ^2^ = 0.43; *d* > 0.80). Schwenk et al.^42^ found a large reduction in fear of falling (*η* ^2^ = 0.30; *d* > 0.80).

## 4 DISCUSSION

This systematic review gathered empirical evidence on digital health interventions for people living with dementia and MCI. The review found diversity in terms of types of interventions, modes of delivery, materials, procedures, location, duration of intervention, length, frequency, and intensity of sessions. As a result, we could only perform two meta‐analyses. The first found a moderate effect size on global cognition. While the effectiveness of cognitive training interventions has been established in the literature,^3^ our meta‐analysis included one intervention delivering exergaming^43^ and one based on brisk walking and behaviour change. Their effectiveness is an addition to the existing evidence^7^ regarding the potential of physical exercise to improve cognitive outcomes. The second meta‐analysis found that digital health interventions are an inferior alternative to the control conditions in the outcome of ADLs. However, the results from the individual studies were inconsistent.

When looking at the characteristics of interventions, supervised training produced larger effect sizes than unsupervised interventions. This finding aligns with a systematic review on face‐to‐face physical activity interventions in non‐cognitively‐impaired older adults that found that supervised balance/resistance training produced larger effect sizes than the unsupervised modality.^57^ In line with previous research,^58^ study findings suggest that supervision can function as a mediating mechanism to maximise participant engagement and adherence with the intervention.

In terms of identifying the most effective interventions, seven interventions produced large effect sizes on any of the outcomes. One intervention only yielded large effect sizes on two outcomes: overall physical and cognitive abilities.^43^ This intervention delivered multiple stepping exergames, requiring participants to start from an upright stance with both feet in the middle of a pressure‐sensitive step training platform, and interact with the game interface by pushing one foot on one of the four different arrows. The device provided real‐time visual, auditory, and somatosensory (vibrating platform) cues, and feedback.

The effectiveness of stepping exergames on physical abilities have been investigated in a previous feasibility study by Garcia et al.^59^ in a sample of older people without cognitive impairment. The authors reported that their step training programme led to improvements in stepping, standing balance, gait speed, and mobility, thus potentially reducing falls. Another study investigated the effects of step exergaming on cognitive abilities (as well as physical ones) of older people,^60^ suggesting that step‐mat training proved effective in reducing fall risk and improving cognitive functions. The promising results of this technology found in this review should warrant further research.

Findings from this review have implications for clinical practice. New digital health interventions should feature some form of “real time” supervision/support. Previous research found that face‐to‐face is the preferred means of interaction for clients, given the added value of direct social contact.^7^ Physiotherapists, Occupational Therapists and Rehabilitation Support Workers also recognise that some rehabilitation activities, particularly risk assessment and progression, are difficult to undertake remotely.^7^ This is further compounded by the inability to use ‘hands on’ techniques to guide practice, posture and support during remote delivery, which is particularly important during falls programmes, commonly accessed by those with cognitive impairment.

However, there are advantages in delivering support remotely, including the possibility to reach clients who live in remote locations or during times of social distancing, and saving on costs/resources when travel is unneeded.^35^, ^61^ Further, among the studies which reported largest effect sizes (on frailty) there was an intervention which left the participants unsupervised and only receiving support remotely through weekly WhatsApp messages.^34^ This potentially shows that a good compromise between effective support and cost efficiency would be a hybrid mix of occasional supervisory face‐to‐face and routine remote support.

This work is characterised by certain strengths. Presenting different digital health service interventions for people living with dementia and Mild Cognitive Impairment can be helpful for e‐intervention developers, enabling them to consult updated evidence on the most effective types of interventions, based on the target population and specific outcomes. In relation to limitations, by focusing only on RCTs/non‐RCTs, we might have missed interventions that have been successfully implemented, but for which effectiveness studies were not produced. Secondly, the great heterogeneity made it impossible to synthesise pooled estimates from all the studies. We advocate that future literature reviews focus on specific types of interventions (e.g., exergaming only) to reduce heterogeneity and facilitate pooling of data. Further, a number of studies reported very large effect sizes, which is quite unusual. This might be due to potential selection bias (e.g., people who agreed to partake in digital health research might be more likely to adhere/comply and obtain benefits), chance (some studies had small samples) and publication bias. Finally, there were also limitations at the review level, such as the use of CASP^25^ for study quality appraisal, which does not attribute a score to reporting of effect size. Effect sizes were not reported in nine studies (28%) and we could only include 13 studies in objective 3.

Finally, the studies did not discuss applicability, accessibility, acceptability, and sustainability, which are key issues for successful digital interventions. Regarding applicability, the diversity of studies included in this review suggests that different interventions may be relevant/ideal for specific subgroups of people living with dementia. For example, an “active” intervention which involves 'exergaming' is very different to a passive intervention such as using telecare sensor mats. Our finding that the digital health interventions produced a negative moderate effect on basic ADLs of participants compared to the control conditions may indicate that assuming that any digital intervention may be beneficial to people living with dementia at different stages of the condition or for a diverse group of individuals (e.g., ethnicity, gender, location, having a live‐in caregiver) will inevitably lead to shortcomings during implementation.

In relation to accessibility, our previous work^7^ found a lack of digital literacy and technology access among users. While some attempts in addressing these issues have been reported,^62^ there still is a need for service design, guidance, and delivery of more dementia‐friendly digital services. Currently, there is contradictory evidence around the acceptability of interventions from older people,^63^ due to concerns around privacy, functionality, doubts around the added value of technology, cost and ease of use of technology, perception of no need for digital solutions, fear of dependence and lack of competency. Acceptability issues can be addressed by involving all prospective client groups in technology development, so that digital services address the real needs of stakeholders.

Regarding sustainability, the impact/uptake of digital health services is rather low, in the lack of fitting infrastructures, inability to find funding, complications with scalability, and uncertainties regarding effectiveness and sustainability.^64^ Current eHealth implementations are usually done post development rather than integrated in the development process. Organisational factors and wider contexts affecting implementation success are therefore often missed.^65^ This risk could be minimised through business modelling at the development stage, by involving potential commercial partners, which can undertake an accurate calculation of costs before they commit to implementing the intervention.^66^ All these key issues warrant careful consideration in future research and service design/implementation.

## 5 CONCLUSION

Digital health interventions can yield positive effects on physical, cognitive, behavioural, and psychological outcomes in people living with MCI and dementia. Stepping exergames were found to generate the largest effect sizes on physical and cognitive abilities. Supervised delivery was linked to greatest benefits, but high costs of face‐to‐face support might make hybrid delivery a better compromise between user's benefits and the limited resources of services. Issues around accessibility, acceptability, and sustainability of digital health interventions for people living with MCI and dementia must be addressed in future research and service development.

## Supporting information

Supporting Information 1

Supporting Information 2

## Data Availability

The data that support the findings of this study are available from the corresponding author upon reasonable request.
