# Supplementary material for: A systematic literature review and meta‐analysis on digital health interventions for people living with dementia and Mild Cognitive Impairment
Source: Int J Geriatr Psychiatry. 2022 May 19;37(6):10.1002/gps.5730. doi: 10.1002/gps.5730 (PMC9321868; doi:10.1002/gps.5730)
Supplement: Supplementary file 2 — Supporting Information 2 [file GPS-37-0-s002.docx]

Appendix B. Search strategy

Medline (ALL) 1946

1. exp Dementia/
2. (dement* or Alzheimer*).mp.
3. 1 or 2
4. exp Telemedicine/
5. exp Telerehabilitation/
6. ("digit* health*" or Ehealth* or Telemedicine or Telerehab* or telehealth* or Mhealth* or "mobile health*" or "Virtual health*" or "Online health*" or "computer-based health*" or "internet-based health*" or "web-based health*" or exergam*).mp.
7. ("Xbox Kinect" or "Wii fit").mp.
8. 4 or 5 or 6 or 7
9. randomized controlled trial.pt.
10. controlled clinical trial.pt.
11. randomized.ab.
12. placebo.ab.
13. clinical trials as topic.sh.
14. randomly.ab.
15. trial.ti.
16. 9 or 10 or 11 or 12 or 13 or 14 or 15
17. exp animals/ not humans.sh.
18. 16 not 17
19. 3 and 8 and 18

Embase 1974 onward

exp dementia/

OR

(dement* or Alzheimer*).mp.

AND

exp telemedicine/

OR

exp telerehabilitation/

OR

("digit* health*" or Ehealth* or Telemedicine or Telerehab* or telehealth* or Mhealth* or "mobile health*" or "Virtual health*" or "Online health*" or "computer-based health*" or "internet-based health*" or "web-based health*" or exergam* or “Xbox Kinect” or “Wii fit”).mp.

AND

Randomized controlled trial/

OR

Controlled clinical trial/

OR

random$.ti,ab.

OR

randomization/

OR

intermethod comparison/

OR

placebo.ti,ab.

OR

(compare or compared or comparison).ti.

OR

((evaluated or evaluate or evaluating or assessed or assess) and (compare or compared or comparing or comparison)).ab.

OR

(open adj label).ti,ab.

OR

((double or single or doubly or singly) adj (blind or blinded or blindly)).ti,ab.

OR

double blind procedure/

OR

parallel group$1.ti,ab.

OR

(crossover or cross over).ti,ab.

OR

((assign$ or match or matched or allocation) adj5 (alternate or group$1 or intervention$1 or patient$1 or subject$1 or participant$1)).ti,ab.

OR

(assigned or allocated).ti,ab.

OR

(controlled adj7 (study or design or trial)).ti,ab.

OR

(volunteer or volunteers).ti,ab.

OR

human experiment/

OR

trial.ti.

NOT

(random$ adj sampl$ adj7 (cross section$ or questionnaire$1 or survey$ or database$1)).ti,ab. not (comparative study/ or controlled study/ or randomi?ed controlled.ti,ab. or randomly assigned.ti,ab.)

OR

Cross-sectional study/ not (randomized controlled trial/ or controlled clinical study/ or controlled study/ or randomi?ed controlled.ti,ab. or control group$1.ti,ab.)

OR

(((case adj control$) and random$) not randomi?ed controlled).ti,ab.

OR

(Systematic review not (trial or study)).ti.

OR

(nonrandom$ not random$).ti,ab.

OR

Random field$.ti,ab.

OR

(random cluster adj3 sampl$).ti,ab.

OR

(review.ab. and review.pt.) not trial.ti.

OR

we searched.ab. and (review.ti. or review.pt.)

OR

update review.ab.

OR

(databases adj4 searched).ab.

OR

(rat or rats or mouse or mice or swine or porcine or murine or sheep or lambs or pigs or piglets or rabbit or rabbits or cat or cats or dog or dogs or cattle or bovine or monkey or monkeys or trout or marmoset$1).ti. and animal experiment/

OR

Animal experiment/ not (human experiment/ or human/)

APA PsycInfo – Ovid

exp Dementia/

OR

(dement* or Alzheimer*).mp.

AND

exp Telemedicine/

OR

exp Telerehabilitation/

OR

("digit* health*" or Ehealth* or Telemedicine or Telerehab* or telehealth* or Mhealth* or "mobile health*" or "Virtual health*" or "Online health*" or "computer-based health*" or "internet-based health*" or "web-based health*" or exergam*).mp.

AND

(“Randomized Controlled Trial” or “Randomised Controlled Trial”).mp.

Cochrane Central Register of Controlled Trials (CENTRAL)

MeSH descriptor: [Dementia] explode all trees

OR

MeSH descriptor: [Alzheimer Disease] explode all trees

OR

dementia or alzheimer*

AND

[Telemedicine] explode all trees

OR

[Telerehabilitation] explode all trees

OR

"digit* NEXT health*" or Ehealth* or Telemedicine or Telerehab* or telehealth* or Mhealth* or "mobile NEXT health*" or "Virtual NEXT health*" or "Online NEXT health*" or "computer-based NEXT health*" or "internet-based NEXT health*" or "web-based NEXT health*" or exergam* or “Xbox Kinect” or “Wii fit”

CINAHL

S1 (MH "Dementia+") OR “dementia”

S2 (MM "Alzheimer's Disease") or "alzheimer's disease"

S3 S1 OR S2

S4 (MH "Telehealth+")

S5 (MH “telemedicine+”)

S6 (MM "Telerehabilitation")

S7 (MM "Exergames")

S8 ("digit* health*" or Ehealth* or Telemedicine or Telerehab* or telehealth* or Mhealth* or mobile health* or Virtual health* or "Online health*" or "computer-based health*" or "internet-based health*" or "web-based health*" or exergam*)

S9 S4 OR S5 OR S6 OR S7 OR S8

S10 MH randomized controlled trials

S11 MH double‐blind studies

S12 MH single‐blind studies

S13 MH random assignment

S14 MH pretest‐posttest design

S15 MH cluster sample

S16 TI (randomised OR randomized)

S17 AB (random*)

S18 TI (trial)

S19 MH (sample size) AND AB (assigned OR allocated OR control)

S20 MH (placebos)

S21 PT (randomized controlled trial)

S22 AB (control W5 group)

S23 MH (crossover design) OR MH (comparative studies)

S24 AB (cluster W3 RCT)

S25 MH animals+

S26 MH (animal studies)

S27 TI (animal model*)

S28 S25 OR S26 OR S27

S29 MH (human)

S30 S28 NOT S29

S31 S10 OR S11 OR S12 OR S13 OR S14 OR S15 OR S16 OR S17 OR S18 OR S19 OR S20 OR S21 OR S22 OR S23 OR S24

S32 S31 NOT S30

S33 S3 AND S9 AND S32

AMED

exp Dementia/

OR

exp Alzheimers disease/

OR

(dementia or alzheimer*).mp.

AND

exp Telemedicine/

OR

("digit* health*" or Ehealth or Telemedicine or Telerehab* or telehealth or Mhealth or mobile health or Virtual health or "Online health" or "computer-based health" or "internet-based health" or "web-based health" or exergam*).mp.

OR

Xbox Kinect.mp.

OR

Wii fit.mp.

Sportdiscus

S1 (DE "ALZHEIMER'S disease") OR (DE "DEMENTIA")

S2 Dementia or alzheimer*

S3 DE "EXERCISE video games" OR DE "NINTENDO Wii Fit games"

S4 "digit* health*" or Ehealth or Telemedicine or Telerehab* or telehealth or Mhealth or "mobile health" or "Virtual health" or "Online health" or "computer-based health" or "internet-based health" or "web-based health" or exergam* or “Xbox Kinect” or “Wii fit”

S5 S1 or S2

S6 S3 or S4

S7 S5 and S6

Web of Science

dementia or alzheimer*

AND

"digit* health*" or Ehealth or Telemedicine or Telerehab* or telehealth or Mhealth or "mobile health" or "Virtual health" or "Online health" or "computer-based health" or "internet-based health" or "web-based health" or exergam* or “Xbox Kinect” or “Wii fit”

AND

"randomised controlled trial" or "randomized controlled trial"

Google Scholar

(dementia OR alzheimer OR alzheimer’s) AND (“digital health” OR “digital healthcare” OR Ehealth OR ehealthcare OR Telemedicine OR Telerehab OR Telerehabilitation OR telehealth OR telehealthcare OR Mhealth OR Mhealthcare OR “mobile health” OR “mobile healthcare” OR “Virtual health” OR “virtual healthcare” OR “online health” OR “online healthcare” OR “computer-based health” OR “computer-based healthcare” OR “internet-based health” OR “internet-based healthcare” OR “web-based health” OR “web-based healtcare” OR exergame OR exergaming OR exergames OR “Xbox Kinect” OR “Wii fit”) AND (“randomised controlled trial” OR “randomized controlled trial”)
